# A Hybrid Genetic-Simulated Annealing Algorithm for the Location-Inventory-Routing Problem Considering Returns under E-Supply Chain Environment

**DOI:** 10.1155/2013/125893

**Published:** 2013-12-29

**Authors:** Yanhui Li, Hao Guo, Lin Wang, Jing Fu

**Affiliations:** ^1^School of Information Management, Central China Normal University, Wuhan 430079, China; ^2^School of Management, Huazhong University of Science and Technology, Wuhan 430074, China; ^3^Department of Industrial and System Engineering, State University of New York at Buffalo, Buffalo, NY 14228, USA

## Abstract

Facility location, inventory control, and vehicle routes scheduling are critical and highly related problems in the design of logistics system for e-business. Meanwhile, the return ratio in Internet sales was significantly higher than in the traditional business. Many of returned merchandise have no quality defects, which can reenter sales channels just after a simple repackaging process. Focusing on the existing problem in e-commerce logistics system, we formulate a location-inventory-routing problem model with no quality defects returns. To solve this NP-hard problem, an effective hybrid genetic simulated annealing algorithm (HGSAA) is proposed. Results of numerical examples show that HGSAA outperforms GA on computing time, optimal solution, and computing stability. The proposed model is very useful to help managers make the right decisions under e-supply chain environment.

## 1. Introduction

The increasing progress of information and prevalence of internet in the 21st century have forced the e-commerce to develop in world-wide rage. In 2012, B2C e-commerce sales grew 21.1% to top $1 trillion for the first time in history of the whole world [1]. Comparing with traditional commerce, customers are liable to return goods under e-commerce environment. Note that many customer returns online accounts for 35% of original orders [2, 3]. Therefore, logistics systems as an important support system in e-commerce need to be adjusted and improved. To adapt to the reality of e-commerce market environment, reverse logistics network and highly integrated logistics process should be the necessities.

Facility location, inventory control, and vehicle routing decisions are critical problems in the design of logistics system. There is much previous work on these three areas. Furthermore, the related work on location and vehicle routing was extended into the field of computer communication and networks [4, 5]. In fact, there is a mutually dependent relationship among these problems in logistics system. Comprehensive optimizing and logistics activities management should be based on this relationship [6]. According to this idea, besides location allocation problem and vehicle routing problem, two-two integration such as location-routing problem (LRP), inventory-routing problem (IRP), and location-inventory problem (LIP) and three integration problem (location-inventory-routing problem, LIRP) start to be researched.

Many papers about the LIP, LRP, and IRP are studied deeply and have made some abundant achievements. However, research on the integration of location-inventory-routing problem is limited. Some researchers strongly appeal to carry out research on LIRP [7, 8]. Liu and Lee [9] firstly proposed the LIRP; they built a model for single merchandise, multi-DPs LRP taking inventory control decisions into consideration and proposed a two-stage heuristic algorithm. In order to avoid being trapped in local optima, Liu and Lin [10] proposed a global optimum heuristic based on the algorithm in the above papers to solve the LIRP. Max Shen and Qi [11] established a nonlinear integer programming model to minimize the total cost that includes location costs, inventory costs, and transportation costs and proposed a Lagrangian relaxation based algorithm to solve the model. Ahmadi Javid and Azad [12] presented an LIRP model in a stochastic supply chain system and established a heuristic method based on a hybridization of tabu search and simulated annealing to solve the LIRP model. Ahmadi-Javid and Seddighi [13] considered the LIRP of a multisource distribution logistics network. A mixed-integer programming formulation was presented and a three-phase heuristic was developed to solve the problem.

Previously, reverse logistics mainly researched independent activities about LIRP; Fleischmann et al. [14] and Jayaraman et al. [15] are interested in determining the location of recycling center with capacity constraints. In recent years, some researches on reverse logistics concerned the integrated system. Lieckens and Vandaele [16] applied a queuing mode in reverse logistics network to solve the facility location problem while considering the impact of inventory costs. Sahyouni et al. [17] developed three generic facility location models that account for both forward and reverse logistics network; Easwaran and Üster [18] proposed a mixed-integer linear programming model to optimize the total cost that consists of location, processing, and transportation costs of the multimerchandise closed-loop supply chains; Srivastava [19] established a reverse logistics network optimization model to optimize the location-distribution problem and capacity decisions, and he pointed out that integrated optimization of processing, storage, transportation, and recycling merchandises is one of the directions of future research.

Previous researches on the reverse logistics system optimization mainly focus on the minimization of the total cost in forward logistics network. To our best knowledge, researches on manufacturing/remanufacturing system by taking customer returns and concept of green logistics recycling into account in reverse logistics are very limited. Since the fact that customers may dissatisfy with merchandise and return it, the cost of processing returns, the cost of inventory and delivery, ordering time, and quantity are changed.

The aim of this study is to develop a practical LIRP model with considering returns under e-supply chain environment and provide a new hybrid heuristic algorithm. To our best knowledge, this work is the first step to introduce returns into the LIRP under e-supply chain environment, which makes it become more practical. We also provide an effective algorithm named hybrid genetic simulated annealing algorithm (HGSAA) to solve this model. Results of numerical examples show that HGSAA outperforms genetic algorithm (GA) on computing time, optimal solution, and computing stability.

The remainder of this paper is organized as follows. In Section 2, a nonlinear integrated programming model based on forward and reverse logistics networks about LIRP is proposed under e-supply chain environment.  Section 3 designs the heuristic algorithm named HGSAA.  Section 4 contains the results of different experiments and corresponding analysis.  Section 5 proposes conclusions and future research directions.

## 2. Mathematical Model

### 2.1. Problem Description

In e-supply chain network, returned merchandises in sales generally have a high integrity, which makes them usually do not need to be repaired and can reenter the sales channels after a simple repackaging process [20]. Therefore, distribution centers and recycling centers can be merged into merchandise centers (MCs). MC is responsible for distributing normal goods to the demand points (DPs) of downstream, meanwhile the returned goods are collected to MCs. After repackaging treatment at MCs, returned goods become resalable normal goods.

Based on the above, the supply chain in this study consists of one plant, multiple MCs, and multiple DPs, which is a three-phases (production base, merchandise centers, and demand points) e-commerce logistics system. Considering the return policy in e-commerce, we optimize system construction, operation of the facility location, inventory control, and coordinate arrangements of vehicle routing.

**Figure 1:**
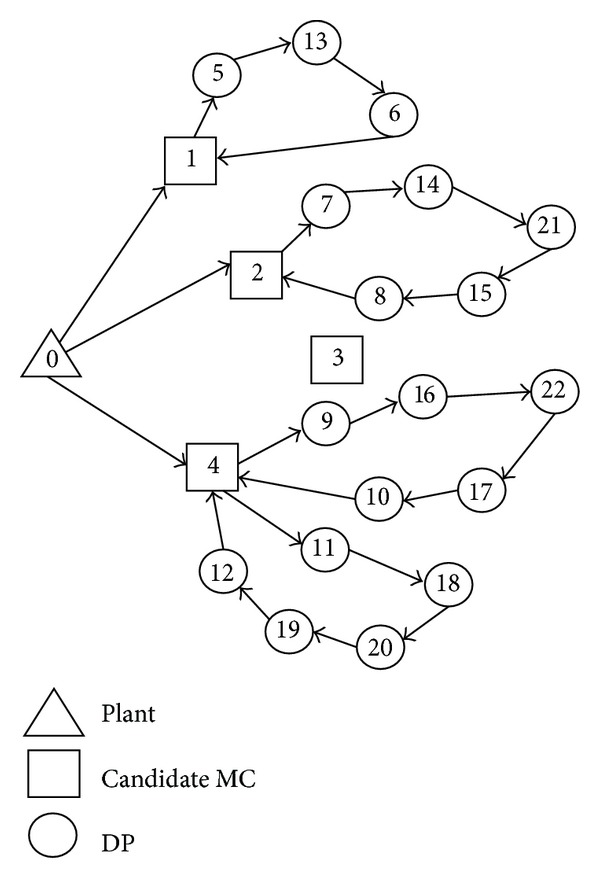
The specific network graph.

The operations of product order and returns are as follows. Previously, the finished productions are transferred from the plant to the MCs. Then the merchandises are delivered to DPs, which in turn collect returned merchandises. Returned merchandises are processed and repackaged in MCs and then sold as normal goods. The operations mode is shown in Figure 1.

The objective of this problem is to determine the quantity, locations, order times, and order size of MCs and arrange the routes that vehicles visiting the DPs in the integrated logistics network. The final target is to minimize the total cost and improve the efficiency of logistics operations. The involved decisions are as follows: (1) location decisions, the optimal number of MCs and their locations; (2) inventory decisions, the optimal order times and order size on a route; (3) routing arrangement, the vehicles deliver merchandises and collect returned merchandises in the order.

### 2.2. Assumptions

1. There is a single type of merchandise.
2. The total demand on each route is less than or equal to the vehicle capacity.
3. The vehicle type is homogeneous.
4. Each route is served by one vehicle.
5. Each route begins and ends at the same MC.
6. The capacity of MCs is infinite.
7. The forward distribution and reverse collection service could be met at the same time.
8. The daily demand and return of each DP are known.
9. The returned merchandises are without quality defect.
10. Returned merchandises are processed and repackaged at MCs.

### 2.3. Model Formulation and Analysis

The cost of MC_*r*_ consists of the following components.

- The annual cost of the dispatching vehicle at MC_*r*_ is given by *N*_*r*_ *e*_*r*_.
- The annual cost of placing an order at MC_*r*_ is given by *N*_*r*_ *f*_*r*_.
- As for the returned merchandise without quality defects, it can be sold again as a normal goods being repackaged; therefore, transportation volume from plant to MC_*r*_ shall deduct the returns ∑_*i*∈**S**_ *q*_*i*_. Thus, the annual transportation cost from plant to MC_*r*_ is given by *λ*∑_*v*∈**V**_∑_*i*∈**S**_ *b*_*r*_(*d*_*i*_ − *q*_*i*_)*Y*_*ir*_^*v*^.
- As the same reason as the third point above, the annual inventory holding costs at MC_*r*_ should consider the returns, too. So, the annual inventory holding cost at MC_*r*_ is given by (*λ*∑_*v*∈**V**_∑_*i*∈**S**_ *h*(*d*_*i*_ + *q*_*i*_)*Y*_*ir*_^*v*^)/2*N*_*r*_. Since there is ∑_*i*∈**S**_(*d*_*i*_ − *q*_*i*_) in component (iii), and here has ∑_*i*∈**S**_(*d*_*i*_ + *q*_*i*_), adding this two parts together, that is ∑_*i*∈**S**_(*d*_*i*_ − *q*_*i*_) + ∑_*i*∈**S**_(*d*_*i*_ + *q*_*i*_) = ∑_*i*∈**S**_ *d*_*i*_. It means the goods flow is equal to demand.
- The annual handling cost at MC_*r*_ is given by *λ*∑_*v*∈**V**_∑_*i*∈**S**_ *c*_*r*_ *d*_*i*_ *Y*_*ir*_^*v*^.
- The annual repackaging cost of returned merchandises at MC_*r*_ is given by *λ*∑_*v*∈**V**_∑_*i*∈**S**_ *pq*_*i*_ *Y*_*ir*_^*v*^.
- The annual total distribution costs from every MC to each DP is given by *N*_*r*_∑_*r*∈**R**_∑_*i*∈**S**^+^_∑_*j*∈**S**^+^_∑_*v*∈**V**_ *ls*_*ij*_ *X*_*ijr*_^*v*^.
- The construction cost of MC_*r*_ is given by ∑_*i*∈**R**_ *a*_*r*_ *Z*_*r*_.

The objective is to minimize the total cost of the system; we formulate the model as follows:

(1) min⁡Z=∑r∈R(er+fr)Nr+λ∑r∈R ∑v∈V ∑i∈Sbr(di−qi)Yirv +λ∑r∈R∑v∈V∑i∈Sh(di+qi)Yirv2Nr  +λ∑r∈R ∑v∈V ∑i∈ScrdiYirv+λ∑r∈R ∑v∈V ∑i∈SpqiYirv +Nr∑r∈R ∑i∈S+ ∑j∈S+ ∑v∈VlsijXijrv+∑r∈RarZr

s.t.

(2)  Zr≥1,   r∈R;

(3)  ∑v∈V ∑r∈R ∑i∈S+Xijrv=1, j∈S;

(4)  ∑v∈V ∑r∈RYirv=1, i∈S;

(5)  ∑j∈S+Xkjrv−∑i∈S+Xikrv=0, k∈S+, v∈V, r∈R;

(6)  ∑i∈S ∑r∈RdiYirv≤g, v∈V;

(7)  Yirv−Zr≤0, r∈R, i∈S, v∈V;

(8)  ∑j∈S+Xijrv+∑j∈S+Xrjrv−Yirv≤1, i∈S, v∈V, r∈R;

(9)  Zr={0,1}, r∈R;

(10)  Yirv={0,1}, r∈R, i∈S, v∈V;

(11)  Xijrv={0,1}, i∈S, j∈S+, r∈R, v∈V,

where the objective function (1) minimizes the system's total cost; (2) ensures at least one MC is established; (3) ensures each DP is served by the only one vehicle which belongs to a certain MC; (4) ensures that each route has only one vehicle; (5) ensures the continuity of delivery routes; (6) ensures vehicle cannot be overloaded; (7) ensures that only the selected MC can carry out distribution services; (8) ensures as long as a route passing through a DP, the corresponding MC would also be on this route; (9)–(11) ensure the integrality of decision variables.

## 3. Solution Approach

In this section, we first give the formula for solving optimal order times *N*_*r*_ and the optimal order size *Q*_*r*_^*v*^. Since calculating *N*_*r*_ and *Q*_*r*_^*v*^ still relies on the decision variables *X*_*ijr*_^*v*^, *Y*_*ir*_^*v*^, and *Z*_*r*_, so we present a heuristic algorithm to get the optimized *X*_*ijr*_^*v*^, *Y*_*ir*_^*v*^, and *Z*_*r*_.

### 3.1. Finding the Optimal Order Times

In the models (1)–(11), the decision variable *N*_*r*_ only has appeared in the objective function. Also, the objective function is convex for *N*_*r*_ > 0. Consequently, we can obtain the optimal value of *N*_*r*_ by taking the derivative of the objective function with respect to *N*_*r*_ as

(12) $$\begin{array}{c} N_{r}=\sqrt{\frac{\sum_{v\in V}\sum_{i\in S}\lambda h\left(d_{i}+q_{i}\right)Y_{ri}^{v}}{2\left(e_{r}+f_{r}+\sum_{i\in S^{+}}\sum_{j\in S^{+}}\sum_{v\in V}ls_{ij}X_{ijr}^{v}\right)}}. \end{array}$$

Then, the optimal order size can be given by

(13) Qr=∑v∈V∑i∈SλdiYrivNr=(∑v∈V∑i∈SλdiYriv×(∑v∈V∑i∈Sλh(di+qi)Yriv2(er+fr+∑i∈S+∑j∈S+∑v∈VlsijXijrv))−1).

### 3.2. Hybrid Genetic Simulated Annealing Algorithm (HGSAA)

The LIRP contains the VRP. As we know, the VRP is an NP-hard problem. This makes LIRP more complicated. It is generally believed that there is no complete, accurate, and not too slow analytic algorithm to solve NP-hand problems. Noting bioinspired computation is widely used for solving optimization problems, we designed a hybrid algorithm based on GA and simulated annealing (SA) to solve the proposed model.

Traditional GA has strong global search ability in solving such problems, but also has defects such as premature and weak local search ability. On the other hand, SA has strong local search ability and no premature problem. Therefore, the combination of GA and SA can overcome the defects of each of the two methods, bring into play their respective advantages, and improve the solving efficiency. This algorithm is named hybrid genetic simulated annealing algorithm (HGSAA).

#### 3.2.1. Relevant Operations of GA

*(1) Encoding.* In a genetic algorithm, a population of candidate solutions (called individuals) to an optimization problem is evolved toward better solutions. Each individual with a set of properties, such as its chromosomes or genotype, can be mutated and altered. Traditionally, solutions are represented in binary as strings of 0 s and 1 s.

This study adopts the natural number coding method; using unrepeatable *R* + *S* natural numbers constitute a sequence, which represents an individual. While 1,2,…, *R* indicate candidate MCs and *R* + 1,…, *R* + *S* indicate DPs, the encoding can describe a candidate solution of the above optimization problem. For example, in Figure 1, the code of the individual corresponding solutions is {1, 5, 13, 6, 2, 7, 14, 21, 15, 8, 3, 4, 9, 16, 22, 17, 10, 11, 18, 20, 19, 12}.

*(2) Fitness Function.* A fitness function is a particular type of objective function that is used to measure the quality of the represented solution. In this study, the fitness function is defined as

(14) $$\begin{array}{c} f_{k}=\frac{1}{Z}. \end{array}$$

*(3) Selection.* During each successive generation, a proportion of the existing population is selected to breed a new generation. Individual solutions are selected through a fitness-based process, where fitter solutions (as measured by a fitness function) are typically more likely to be selected. Wheel selection operator [21] (also known as proportional selection operator) is used. Suppose the population size is *N*, the fitness value of the individual *k* is *f*_*k*_, a number *ξ* ∈ [0, 1] is generated randomly. If (∑_*k*=1_^*i*−1^ *f*_*k*_/∑_*k*=1_^*N*^ *f*_*k*_) < *ξ* ≤ (∑_*k*=1_^*i*^ *f*_*k*_/∑_*k*=1_^*N*^ *f*_*k*_) then individual *i* is selected to be replicated.

Because this method has great randomness in the selection of individuals, the simulated annealing algorithm with faster local convergence is added to the GA to increase the convergence speed in selection operation. 

*(4) Crossover.* Crossover is a process of taking more than one parent individuals and producing a child individual from them. Crossover is used to vary the programming of a chromosome or chromosomes from one generation to the next. Partially matched crossover (PMX) [22] is used in this paper.

**Step 1**

Select two parent individuals randomly from the population;

**Step 2**

generate two random cut points to represent the mapped segments;

**Step 3**

exchange the segments of the two parent individuals to produce two new individuals;

**Step 4**

determine the mapping relations between two segments;

**Step 5**

legalize two new individuals with mapping relationship through repair strategy.

*(5) Mutation.* Mutation is used to maintain genetic diversity from one generation of a population of individuals to the next. The purpose of mutation in GAs is preserving and introducing diversity. Mutation should allow the algorithm to avoid local minima by preventing the population of individuals from becoming too similar to each other, thus slowing or even stopping evolution. A simple and efficient mutation operation, that is, swap mutation [23], is used. The details are as follows.

**Step 1**

Select one parent individual randomly from the population;

**Step 2**

generate two random numbers to represent the mutation points;

**Step 3**

swap the positions of these two mutation points to produce a new individual.

Compared with other mutations, studies show that convergence rate of this method has a greater advantage in population control. It can effectively prevent premature convergence of GA and avoid the occurrence of local optimal solution.

#### 3.2.2. Relevant Operations of SA

*(1) The Annealing Process to Accept the New Individual.* In order to prevent the population into local optimization, the Metropolis acceptance criteria in SA are applied into the GA in this paper. We reserved the best parent individual in a population named old, and then selected the best offspring individual in another population named new; old and new go into the next generation population through competition. Let Δ_*f*_ = *f*_new_ − *f*_old_, if Δ_*f*_ < 0, then the individual *new* is received, and hold it to the next generation; otherwise, the individual new is received with the probability *p* = exp⁡(−Δ_*f*_/*t*) > random  digit, where *t* is annealing temperature.

*(2) Temperature Amended Criterion.* One of the key steps in the process of SA is to determine the update function of temperature; the function is used to continuously reduce the temperature value, when its temperature is reduced to approximately zero, the final solution is considered as the global optimal solution. The update function is *t*_*k*+1_ = *αt*_*k*_, *k* ≥ 0, 0 < *α* < 1; the nearer *α* is to 1, the slower the temperature decreases.

#### 3.2.3. Termination

Commonly, the algorithm terminates when either a maximum number of generations have been produced, or a satisfactory fitness level has been reached for the population. In this paper, the termination condition is that the fitness has reached a plateau such that successive *M* iterations no longer produce better results.

#### 3.2.4. Algorithm Flow

**Step 1**

Set the initial parameters: coordinates of the DPs and the candidate MCs, demands and returns of the DPs, the maximum capacity of the vehicle *g*, the population size *N*, evolution terminate generation *M*, crossover probability *p*_*c*_, mutation probability *p*_*m*_, temperature of the cooling coefficient *α*, the initial annealing temperature *T*_0_, and so on.

**Step 2**

Calculate the fitness value of an individual. If the parent optimal solution and offspring optimal solution are equal during continuous *M* generations, the algorithm stops and outputs the current optimal solution; otherwise, go to the next step.

**Step 3**

Perform individual selection, crossover, and mutation operations, generate new population, and calculate the fitness value.

**Step 4**

If *f*_*i*_ < *f*_*j*_  (*j* > *i*), accept the new individual; otherwise, accept the new individual with the probability *p* = exp⁡(−Δ_*f*_/*t*);

**Step 5**

Update the annealing temperature and return to Step 2.

The pseudocodes of HGSAA are shown in Pseudocode 1.

**Pseudocode 1:**
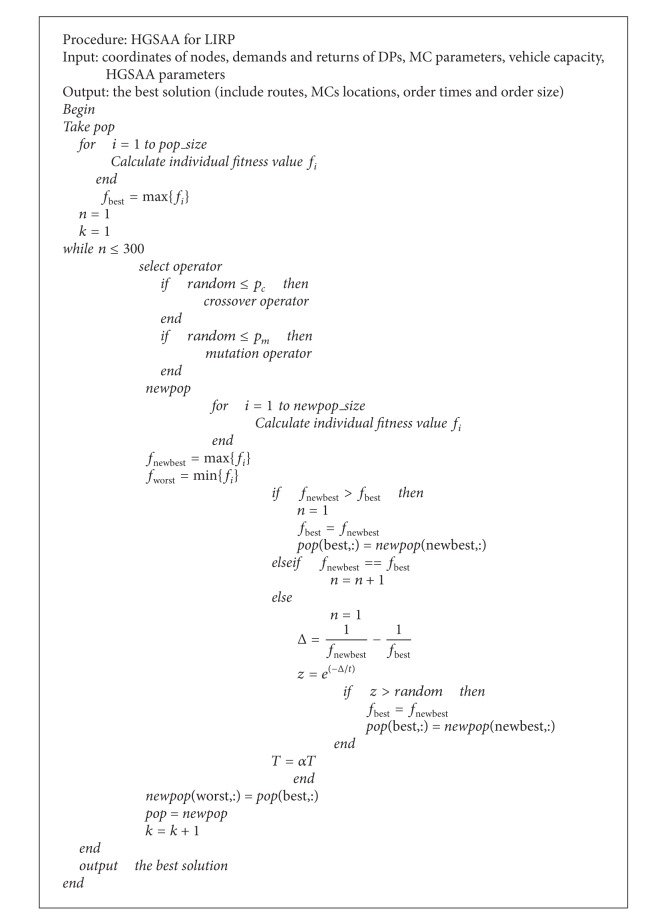
Pseudocode of the proposed HGSAA.

## 4. Computational Experiments and Results Analysis

### 4.1. An Example

An example is used to illustrate the proposed heuristic method. The data of Gaskell 67-29×5 come from the LRP database at University of Aveiro [24]. Gaskell 67 is the name of this instance; 29×5 means there are 29 DPs and 5 candidate MCs. The coordinate of all nodes and the demands of DPs are given by the database. To facilitate the calculation, the daily demands of DPs are set as 1/25 of corresponding demands in the LRP database. The other data are as follows: the inventory holding cost per unit of merchandise per year *h* = 5; the vehicle capacity *g* = 500; the delivering cost per unit distance *l* = 1; the handling cost per unit product at MC_*r*_ *c*_*r*_ = 4; fixed cost of dispatching vehicles per time at MC_*r*_ *e*_*r*_ = 18; the conversion constant *λ* = 300; repackaging cost of unit returned merchandise *p* = 3; *q*_*i*_, *b*_*r*_, *f*_*r*_ are uniformly generated from *U*[1,5], *U*[6,10], *U*[16,20].

According to the experience of literatures [25, 26], the related parameters of the HGSAA are set as follows: the population size *N* = 20; crossover probability *p*_*c*_ = 0.8; mutation probability *p*_*m*_ = 0.001; evolution terminate generation *M* = 300; initial temperature *T*_0_ = 100; temperature cooling coefficient *α* = 0.9.

**Figure 2:**
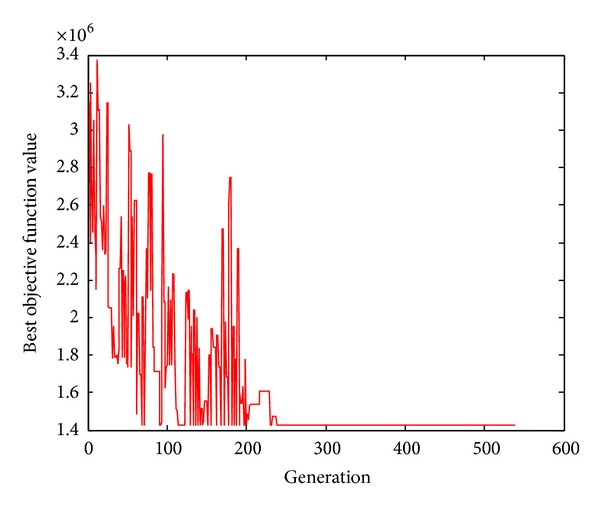
Trends of optimal objective function value by HGSAA.

Based on Matlab 6.5 platform, we programmed the HGSAA and then run it 30 times on a computer (CPU: Intel Core 2 Duo P8400 @ 2.26 GHz 2.27 GHz; RAM: 3 GB DDR; OS: Windows Vista). One of the minimum values of objective function is 1404100; the individual is encoded as {5, 3, 34, 23, 21, 33, 11, 32, 4, 12, 28, 19, 29, 14, 25, 13, 22, 27, 7, 15, 26, 8, 6, 18, 30, 2, 1, 10, 20, 24, 31, 9, 16, 17}, and *N*_*r*_ = {56, 0, 46, 94, 0}, *Q*_*r*_^*v*^ = {333, 231, 444, 300, 409, 368}.  Figure 2 shows the trends of optimal objective function values along with the evolution generations.  Table 1 shows the solution.

For comparison, GA is programmed by Matlab 6.5 as well, and the instance Gaskell 67 was run 30 times on the same computer. The optimal objective function values of these two algorithms are shown in Table 2, the CPU time for calculation is shown in Table 3.

**Figure 3:**
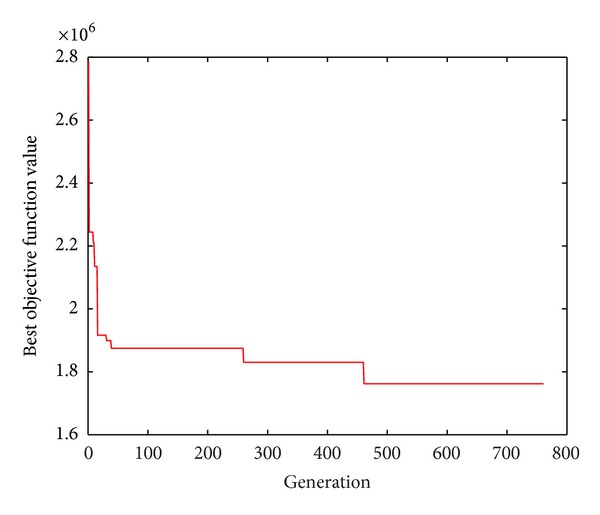
Trends of optimal objective function value by GA.

Figure 3 shows the trends of optimal objective function value along with the evolution generations by GA.

**Figure 4:**
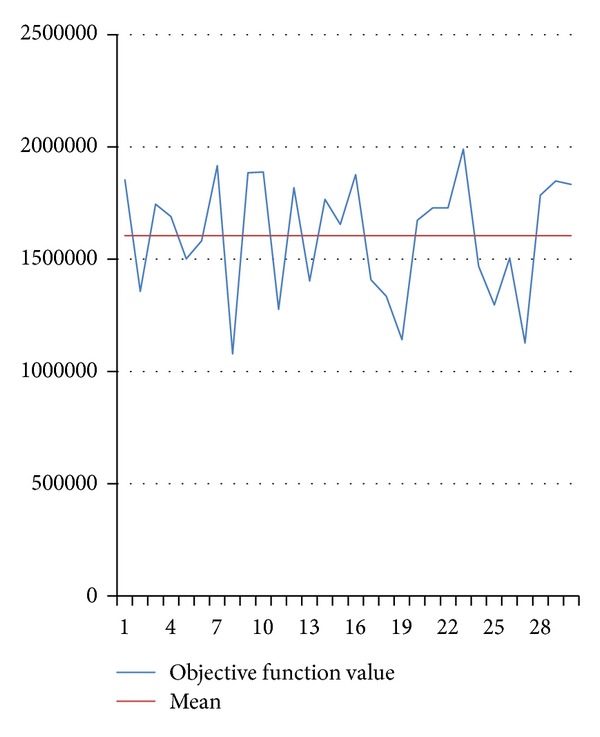
The fluctuation curve of optimal objective function value by HGSAA.

**Figure 5:**
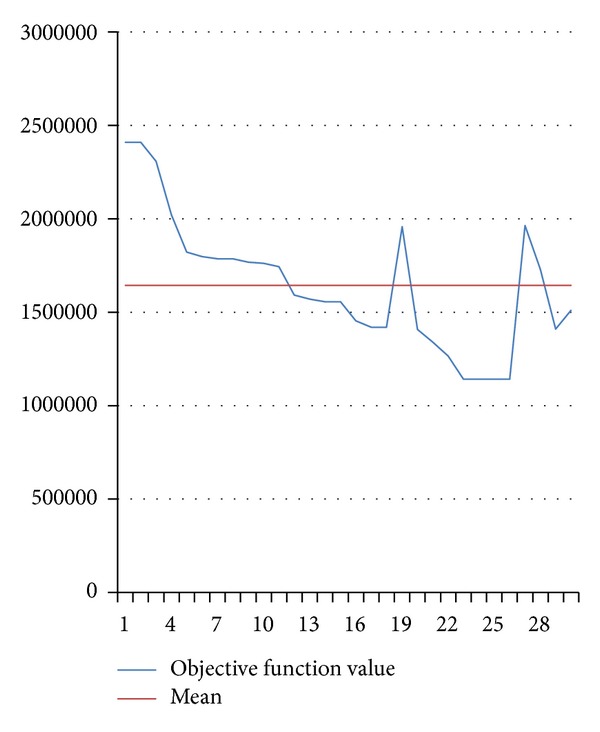
The fluctuation curve of optimal objective function value by GA.

The fluctuation curves of optimal objective function values in 30 times are shown in Figures 4 and 5, respectively.

Figures 2 and 3 show that HGSAA can converge to the optimal solution more quickly than GA. Moreover, HGSAA has better stability than GA, which can easily be found from Tables 2 and 3 and Figures 4 and 5.

### 4.2. Extended Experiments

In this section, a series of experiment is given to show that HGSAA is more efficient and stable than GA. Similarly as Section 4.1, all the experiments in this section come from LRP database of the University of Aveiro [24]. In order to ensure the demands of DPs are not more than the vehicle capacity, we need to enumerate some instances. In this study, the daily demands are set as 1/15 of corresponding demands of Gaskell 67-22×5.

Results of numerical example in Section 4.1 show that the related parameters of HGSAA in Gaskell 67-22×5 are reasonable. Thus, we employ these parameters in the remainder of this section. Each instance was calculated 30 times by HGSAA and GA, respectively; the results are shown in Tables 4 and 5.

Table 4 shows that HGSAA can obtain better objective function value than GA.  Table 5 shows that HGSAA takes less time to achieve the optimal solution than GA. Results of Tables 4 and 5 show that HGSAA is more stable than GA.

## 5. Conclusion and Future Research

Under the e-commerce environment, customers have a higher return rate. At the same time, the returned goods have generally no quality defect and with great integrity. Just after a simple repackaging process, the returned goods can reenter the sales channels, which put forward high requirements to the logistics system that support the operation of e-commerce. This study handles the above interesting problem and provides an effective heuristic. The main contributions are as follows.

1. In reality, the cost of processing returned merchandises is produced considering the customers are not satisfied with products and maybe return them. We firstly design a LIRP model to minimize the total cost produced by both forward and reverse logistics networks. It is very useful to help managers make the right decision under e-supply chain environment.
2. An integration LIRP model with returns is an NP-hard problem and very hard to be solved by analytical method. So, a heuristic algorithm named HGSAA is designed by integrating GA with SA.
3. Results of experimental data show that HGSAA outperforms GA on computing time, optimal solution, and computing stability. HGSAA is a good candidate to solve the proposed LIRP model effectively.

However, some extensions should be considered in further work. Considering the variety of the types of products and service vehicles, the multiple products and multiple vehicles type model should be established. In reality, decision makers are always in front of imprecise and vague operational conditions [27]. Uncertainties have been tackled in a lot of ways and fuzzy set theory has a long history for handle imprecise values [28]. Considering the fuzzy demand of customs or related fuzzy costs, more practical LIRP model should be developed. Moreover, differential evolution algorithms (DEs) have turned out to be one of the best evolutionary algorithms in a variety of fields [29]. In the future, we may use an improved DE to find better solutions for the LIRPs. The integration research and practice of the management of e-commerce logistics system can be constantly improved.

## Figures and Tables

**Table 1:** Solution of Gaskell 67-29×5.

MC	Routing number	Routing	Order times	Order quantity
1	1	*i*_10_-*i*_20_-*i*_24_-*i*_31_-*i*_9_	56	333
	2	*i*_16_-*i*_17_	56	231
3	3	*i*_34_-*i*_23_-*i*_21_-*i*_33_-*i*_11_-*i*_32_	46	444
4	4	*i*_12_-*i*_28_-*i*_19_-*i*_29_-*i*_14_-*i*_25_-*i*_13_-*i*_22_-*i*_27_	94	300
	5	*i*_7_-*i*_15_	94	409
	6	*i*_26_-*i*_8_-*i*_6_-*i*_18_-*i*_30_	94	368

**Table 2:** Statistical results of optimal objective function value of two algorithms.

	Maximum	Minimum	Mean	Standard deviation	Coefficient of variation
HGSAA	1989900.00	1078700.00	1605543.33	262462.64	0.16
GA	2410900.00	1142000.00	1644678.00	353993.10	0.22

**Table 3:** Statistics results of CPU time for calculation of two algorithms.

	Maximum	Minimum	Mean	Standard deviation	Coefficient of variation
HGSAA	5.93	3.35	4.68	0.80	0.17
GA	7.35	3.63	4.93	1.18	0.24

**Table 4:** Optimal objective function values of two algorithms.

Instance name	Algorithm	Maximum	Minimum	Mean	Standard deviation	Coefficient of variation
Perl 183-12×2	HGSAA	740420	157810	502684	176218.7	0.350555
	GA	1322000	219800	660282	234416.0	0.355024
Gaskell 67-22×5	HGSAA	1718000	1071600	1432110	213118.8	0.148815
	GA	3146500	1506200	2170660	553894.4	0.255173
Gaskell 67-36×5	HGSAA	3635500	1877000	2879510	561628.4	0.195043
	GA	3836900	2219400	2886460	582830.7	0.201919
Perl 183-55×15	HGSAA	4120700	3606800	3985110	152566.1	0.038284
	GA	4307000	3755100	4090590	185978.4	0.045465
Christofides 69-75×10	HGSAA	5562400	4290900	4826600	394532.4	0.081741
	GA	6359300	4859800	5418878	525869.4	0.097044
Perl 83-85×7	HGSAA	6529200	5050500	5693300	531113.4	0.093287
	GA	7057200	5545800	6283210	428004.8	0.068119
Christofides 69-100×10	HGSAA	5978900	5074600	5592260	332403.7	0.05944
	GA	6177500	5211900	5792190	354180.4	0.061148

**Table 5:** CPU time (seconds) for calculation of two algorithms.

Instance name	Algorithm	Maximum	Minimum	Mean	Standard deviation	Coefficient of variation
Perl 183-12×2	HGSAA	0.8424	0.4992	0.65156	0.085708	0.131543
	GA	0.9672	0.5304	0.6696	0.102803	0.153529
Gaskell 67-22×5	HGSAA	1.716	1.092	1.37436	0.169531	0.123353
	GA	2.1216	1.17	1.55688	0.272075	0.174756
Gaskell 67-36×5	HGSAA	3.7596	2.2776	3.2058	0.501429	0.156413
	GA	4.8984	2.5584	3.81108	0.760696	0.199601
Perl 183-55×15	HGSAA	12.0121	8.5957	9.91249	0.999262	0.100808
	GA	12.6517	9.8125	10.91837	0.846487	0.077529
Christofides 69-75×10	HGSAA	13.3225	10.1713	11.74674	1.162717	0.098982
	GA	13.7437	10.2805	12.30382	1.370948	0.111425
Perl 83-85×7	HGSAA	21.6373	17.0509	18.94194	1.305246	0.068908
	GA	24.3518	17.8621	21.04736	2.279007	0.10828
Christofides 69-100×10	HGSAA	33.2906	30.1859	31.36279	0.93519	0.029818
	GA	33.5558	30.6386	32.21258	0.962075	0.029866

## Notations

### Sets

**R**: Set of candidate MC
**S**: Set of DPs
**S**^+^: Set of candidate MCs and DPs; that is, **S** ^+^ = **S** ∪ **R**
**V**: Set of routes are available for the routing from the MCs to DPs.

### Constants

*a*_*r*_: Fixed (annual) administrative and operational cost of MC_*r*_
*b*_*r*_: The unit of product transportation cost from plant to MC_*r*_
*c*_*r*_: The handling costs per unit product at MC_*r*_
*d*_*i*_: Mean (daily) demand at DP_*i*_
*e*_*r*_: Fixed cost of dispatching vehicles per time at MC_*r*_
*f*_*r*_: Fixed cost of placing an order at MC_*r*_
*g*: The vehicle capacity
*h*: The inventory holding cost per unit of merchandise per year
*l*: The delivering cost per unit distance
*p*: Repackaging cost of unit returned merchandise at MC
*q*_*i*_: The quantity of merchandises returned by DP_*i*_ per day
*s*_*ij*_: The distance from depot *i* to depot *j*
*λ*: Working days per year.

### Decision Variables

*N*_*r*_: Optimal order times at MC_*r*_
*Q*_*r*_^*v*^: Optimal order size on routing *v* for MC_*r*_
*X*_*ij**r*_^*v*^: 1, if depot *j* is from depot *i* served by a MC_*r*_ on routing *v*, and 0 otherwise
*Y*_*ir*_^*v*^: 1, if DP_*i*_ is assigned to MC_*r*_ on routing *v*, and 0 otherwise
*Z*_*r*_: 1, if candidate MC_*r*_ is selected as a MC location, and 0 otherwise.
